# Predicting the grade of meningiomas by clinical–radiological features: A comparison of precontrast and postcontrast MRI

**DOI:** 10.3389/fonc.2022.1053089

**Published:** 2022-12-01

**Authors:** Yuan Yao, Yifan Xu, Shihe Liu, Feng Xue, Bao Wang, Shanshan Qin, Xiubin Sun, Jingzhen He

**Affiliations:** ^1^ Department of Radiology, Qilu Hospital of Shandong University, Jinan, Shandong, China; ^2^ Department of Radiology, The First Affiliated Hospital of Shandong First Medical University and Shandong Provincial Qianfoshan Hospital, Jinan, Shandong, China; ^3^ Department of Radiology, The Affiliated Hospital of Qingdao University, Qingdao, Shandong, China; ^4^ Department of Biostatistics, School of Public Health, Cheeloo College of Medicine, Shandong University, Jinan, Shandong, China

**Keywords:** meningioma, precontrast MRI, WHO grade, nomogram, radiological prediction

## Abstract

**Objectives:**

Postcontrast magnetic resonance imaging (MRI) is important for the differentiation between low-grade (WHO I) and high-grade (WHO II/III) meningiomas. However, nephrogenic systemic fibrosis and cerebral gadolinium deposition are major concerns for postcontrast MRI. This study aimed to develop and validate an accessible risk-scoring model for this differential diagnosis using the clinical characteristics and radiological features of precontrast MRI.

**Methods:**

From January 2019 to October 2021, a total of 231 meningioma patients (development cohort n = 137, low grade/high grade, 85/52; external validation cohort n = 94, low-grade/high-grade, 60/34) were retrospectively included. Fourteen types of demographic and radiological characteristics were evaluated by logistic regression analyses in the development cohort. The selected characteristics were applied to develop two distinguishing models using nomograms, based on full MRI and precontrast MRI. Their distinguishing performances were validated and compared using the external validation cohort.

**Results:**

One demographic characteristic (male), three precontrast MRI features (intratumoral cystic changes, lobulated and irregular shape, and peritumoral edema), and one postcontrast MRI feature (absence of a dural tail sign) were independent predictive factors for high-grade meningiomas. The area under the receiver operating characteristic (ROC) curve (AUC) values of the two distinguishing models (precontrast–postcontrast nomogram vs. precontrast nomogram) in the development cohort were 0.919 and 0.898 and in the validation cohort were 0.922 and 0.878. DeLong’s test showed no statistical difference between the AUC values of the two distinguishing models (*p* = 0.101).

**Conclusions:**

An accessible risk-scoring model based on the demographic characteristics and radiological features of precontrast MRI is sufficient to distinguish between low-grade and high-grade meningiomas, with a performance equal to that of a full MRI, based on radiological features.

## Introduction

Meningioma is one of the most common types of intracranial neoplasm, accounting for approximately one-third of all central nervous system tumors (1). According to the World Health Organization (WHO), most meningiomas are benign (Grade I), while 20%–30% are high grade, divided into Grade II (atypical meningioma) and Grade III (anaplastic meningioma), which have a worse prognosis and higher tendency of recurrence as compared to benign meningiomas (2). Because treatment methods for low-grade and high-grade meningiomas differ significantly, it is usually advisable to separate them before proceeding with biopsy or resection.

Magnetic resonance imaging (MRI), especially postcontrast MRI, is a clinical routine for the diagnosis and preoperative evaluation of intracranial meningiomas (3). However, the use of gadolinium-based contrast agents contributes to the cost of MRI and prolongs image-acquisition time. Additionally, these contrast agents may be a contraindication among patients who have renal dysfunctions, as nephrogenic systemic fibrosis may occur among them (4). Furthermore, there are also concerns regarding gadolinium deposition, particularly in patients who require frequent follow-ups (5). Therefore, identifying high-grade meningiomas based on precontrast MRI is crucial under these circumstances. Some studies have explored the value of non-contrast advanced MRI, such as amide proton transfer imaging (6) or intra-voxel incoherent motion imaging (7), in identifying high-grade meningiomas and have achieved good performance. However, these advanced MRI techniques may not always be available, especially for primary healthcare institutions. Evaluation based on commonly available precontrast MRI has a wider universality.

Recently, radiomics and machine learning with commonly used MRI data have been proven qualified enough to separate high-grade tumors from low-grade ones (8). However, all studies associated with meningiomas used both precontrast and postcontrast MRI data. In addition, although these methods are effective, some inherent disadvantages may restrict their scope of utilization. Firstly, these low-level features extracted could not be easily explained by pathophysiological knowledge and not be easily accepted by radiologists. Secondly, signal heterogeneity of MRI data acquired from varied MR machines restricts the robustness and generalizability of the trained classification models. Conventional radiological features are the ones acquired from visual evaluation by radiologists and explainable by pathophysiological knowledge and are also insensitive to signal heterogeneity of MRI data. Lastly, a risk-scoring model is more practical than the trained machine learning models in clinical practice. Therefore, radiological features and a simple risk-scoring model would be the preferred and urgent method in clinical practice.

To our knowledge, the distinguishing performance of the risk-scoring model for meningiomas based on the radiological features from precontrast MRI has not been explored yet. Hence, the aim of this retrospective study is to develop and validate an accessible risk-scoring nomogram model for distinguishing high-grade meningiomas from low-grade ones using clinical characteristics and radiological features from commonly available precontrast MRI. This work would facilitate the risk stratification and clinical decision-making of meningiomas.

## Materials and methods

### Study population

**Figure 1:**
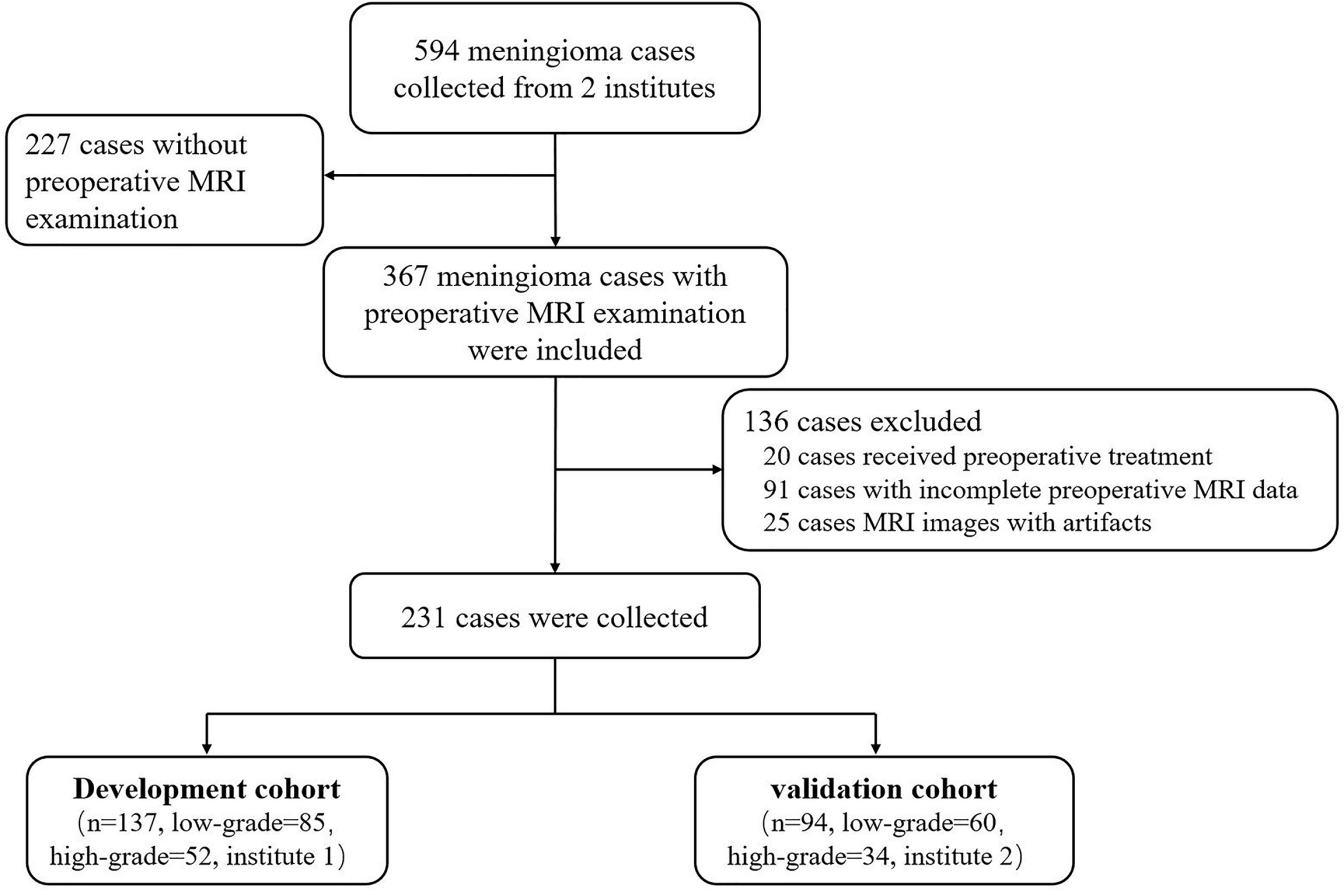
How the study population was collected.

A total of 594 patients with histologically confirmed meningiomas were retrospectively reviewed from two institutions. Patients diagnosed in Hospital 1 from January 2019 to October 2021 were grouped into the development cohort, and patients diagnosed in Hospital 2 from February 2018 to March 2021 were sorted into the validation cohort. The grade of meningiomas was diagnosed based on *the 2021 World Health Organization Classification of Tumors of the Central Nervous System* (9). The exclusion criteria were as follows: 1) patients who had received any preoperative treatment, 2) patients who had incomplete preoperative MRI examinations, and 3) unqualified MRI data due to the presence of artifacts. A total of 231 patients were included in this study. For anaplastic meningiomas (Grade III), we only found four patients in the development cohort and two patients in the validation cohort who met the above-mentioned requirements, the number of which were too few to be statistically significant. Therefore, Grade II and III meningiomas were classified as one group in order to have a comparable subgroup. Grade I meningiomas were treated as low grade and Grade II and III meningiomas as high grade. Finally, 137 patients (55.80 ± 11.14 years old; male/female, 38/99; low grade/high grade, 85/52) in the development cohort and 94 patients (54.73 ± 13.23 years old; male/female, 37/57; low grade/high grade, 60/34) in the validation cohort were included in this retrospective study. How the study population was collected is presented in **Figure 1**.

This study was approved by the Institutional Review Board, and the requirement for patient approval or informed consent was waived owing to the retrospective nature of the study. All the experiments were performed in accordance with the ethical standards established in the 1964 Declaration of Helsinki and its later amendments.

### MRI acquisition

All patients were imaged in the supine position with a 3.0-T MRI machine (Philips Achieva for the development cohort and GE Discovery 750 for the validation cohort) using a receive quadrature 20-channel head-and-neck coil, and the imaging protocol was the same for all patients in both institutions. The MR examination is a routine protocol for brain examination, including axial T1-weighted imaging (T1WI), axial T2-weighted imaging (T2WI), axial diffusion-weighted imaging (DWI), axial fluid-attenuated inversion recovery (FLAIR), and axial, sagittal, and coronal postcontrast T1WI. The MR scan protocols are provided in the **Supplementary Material**.

### Definition of radiological features of meningiomas

Two experienced radiologists (HJZ and YY, with 12 and 5 years’ experience, respectively) who were blinded to the histopathological diagnosis evaluated the radiological features of MRI by consensus reading. The definition of radiological features of meningiomas is described in **Table 1**.

1) Tumor shape: categorized as regular, lobulated, and irregular. Irregular shapes included meningiomas with infiltrating margins and tumors that cannot be classified as round or lobulated.
2) T2 signal intensity: compared to the intensity of the normal gray matter.
3) Tumor–brain interface was categorized as follows: tumors with cerebrospinal fluid (CSF) clefts or distinct low-intensity borders were regarded as “clear” tumor–brain interface, whereas tumors without an obvious demarcation from the adjacent brain tissue were regarded as “unclear”.
4) Capsular enhancement was categorized as positive or negative, depending on whether there was an entire enhanced layer around the tumor.
5) Calculation of the apparent diffusion coefficient (ADC) values was performed using the software programs available on workstations provided by the corresponding manufacturer (Viewforum workstations, Philips and Leonardo workstations, Siemens, Munich, Germany). Three round regions of interest (ROIs), varying from 15 to 30 mm^2^ in size, were placed manually in the solid part of the tumor, avoiding any cystic or calcified regions. Each ROI was separately placed in the three darkest regions on the ADC maps, signifying the areas with the highest cellular density in the tumor. Then, the mean tumor ADC values (ADC_T_) were calculated. In order to acquire control ADC values (ADC_C_), 20–30 mm^2^ round ROIs were also drawn on the contralateral normal-appearing white matter unaffected by the tumor. The ADC_T_/ADC_C_ ratios were calculated to obtain normalized ADC values (nADC).

**Table 1:** The definition of radiological features of meningiomas.

MRI features	Category or definition
Tumor location	Convexity, skull base, falx, posterior fossa, other included ventricle, and extra cranium
Volume of tumor (mm**^3^**)	Volume = maximum AP × maximum ML × maximum SI
Tumor shape	Regular, lobulated, irregular
Signal feature	Heterogeneous, homogeneous
T2 signal intensity	Hypointense, isointense, hyperintense
Intratumoral cystic change	Yes, no
Peritumoral brain edema	Maximum diameter of the hyperintense brain parenchyma surrounding the tumor
Tumor–brain interface	Clear, unclear
Contrast enhancement	Homogeneous, heterogeneous
Capsular enhancement	Positive, negative
Dural tail sign	Positive, negative
nADC	nADC = ADC_T_/ADC_C_

AP, anteroposterior diameter; ML, mediolateral diameter; SI, suprainferior diameter.

### Clinicoradiologic model and nomogram construction

The differences in demographic data and radiological features between low-grade and high-grade meningiomas in the development cohort were tested first to determine the distinguishing features. Two types of multivariate logistic regression models were built to explore the distinguishing performance of combined selected features. The first logistic regression analysis (precontrast–postcontrast model) was performed using all the above-selected demographic and radiological features (including precontrast and postcontrast MRI features). Next, only selected radiological features from precontrast MRI and demographic features were included in the second logistic regression model (precontrast model). The odds ratios (ORs), 95% confidence intervals (CIs), and *p*-values of each independent factor for both logistic regression models were calculated. The nomograms for the two models were presented separately.

Finally, the external validation cohort was used to confirm the robustness and generalizability of the two models. The models also used the bootstrap method for internal validation during the training, with 1,000 bootstrap replicates.

### Statistical analyses

The chi-squared test and Fisher’s exact test were used to investigate group differences in categorical data. Student’s t-test for normally distributed continuous variables and the Mann–Whitney U test for variables with skewed distributions were performed to explore the differences between the two groups. Receiver operating characteristic (ROC) curves, precision-recall curves (PR curves), and calibration curves were used to assess the classifying performance of the two logistic regression models. Sensitivity, specificity, and area under the curve (AUC) derived from ROC curves were described. DeLong’s test was used to compare the ROC curves. Decision curve analysis (DCA) was implemented to determine the clinical practicability of the two nomograms based on the net benefits at different threshold probabilities. For all tests, a two-sided *p*-value <0.05 was considered significantly different. Statistical analyses were made by SPSS 25.0 version for Windows and R statistical software (version 4.2.0, https://www.r-project.org).

## Results

### Characteristics of study populations

A total of 231 patients were included in this study: 215 patients (93.1%) were symptomatic, and 16 patients (6.9%) were asymptomatic. The characteristics of the study population of both the development cohort and validation cohort are summarized in **Table 2**. None of the demographic and radiological features were found to be statistically different between the two groups.

**Table 2:** Characteristics of patients in the development and validation cohorts.

	Development cohort (n = 137)	Validation cohort (n = 94)	*p*-Value
**Age in years**	55.80 ± 11.14	54.73 ± 13.23	0.401
**Sex**			
Male	38 (27.7%)	37 (39.4%)	0.064
Female	99 (72.3%)	57 (60.6%)	
**Tumor location**			
Convexity	51 (37.2%)	40 (42.6%)	0.435
Skull base	37 (27.0%)	26 (27.7%)	
Falx	24 (17.5%)	12 (12.8%)	
Posterior fossa	20 (14.6%)	15 (16.0%)	
Other	5 (3.6%)	1 (1.1%)	
**Volume (×10^4^ mm^3^)**	4.70 (1.68–9.37)	3.78 (1.36–9.97)	0.826
**Shape**			
Regular	75 (54.7%)	42 (44.7%)	0.195
Lobulated	30 (21.9%)	30 (31.9%)	
Irregular	32 (23.4%)	22 (23.4%)	
**T2 signal feature**			
Heterogeneous	88 (64.2%)	60 (63.8%)	0.950
Homogeneous	49 (35.8%)	34 (36.2%)	
**T2 signal intensity**			
Hypointense	4 (2.9%)	7 (7.4%)	0.141
Isointense	18 (13.1%)	17 (18.1%)	
Hyperintense	115 (83.9%)	70 (74.5%)	
**Cystic change**			
Yes	40 (29.2%)	26 (27.7%)	0.799
No	97 (70.8%)	68 (72.3%)	
**Brain edema (mm)**	4.60 (0.00–23.55)	0.93 (0.00–27.65)	0.304
**Tumor–brain interface**			
Clear	136 (99.3%)	90 (95.7%)	0.177
Unclear	1 (0.7%)	4 (4.3%)	
**nADC**	1.05 (0.96–1.20)	1.05 (0.95–1.24)	0.865
**Contrast enhancement**			
Heterogeneous	94 (68.6%)	61 (64.9%)	0.554
Homogeneous	43 (31.4%)	33 (35.1%)	
**Capsular enhancement**			
Present	25 (18.2%)	20 (21.3%)	0.568
Not present	112 (81.8%)	74 (78.7%)	
**Dural tail sign**			
Present	101 (73.7%)	71 (75.5%)	0.757
Not present	36 (26.3%)	23 (24.5%)	

### Demographic and radiological features

For demographic data, sex distribution was significantly different between low-grade and high-grade meningiomas, in which male patients have a higher possibility to suffer from high-grade meningiomas (*p* = 0.028). In addition, female patients had a higher incidence in both groups (78.8% and 61.5% for low-grade and high-grade meningiomas, respectively).

For precontrast radiological features, bigger tumor volume (*p* < 0.001) with lobulated or irregular shape (*p* < 0.001) and heterogeneous signal intensity on T2WI (*p* = 0.002) were associated with a higher risk for high-grade meningiomas. Tumors with cystic changes (*p* < 0.001) and larger brain edema (*p* < 0.001) were more often in high-grade meningiomas in our cohort. The relation of meningiomas tumor to venous sinus is different for low-grade and high-grade meningiomas (*p* = 0.041). The high-grade tumors were more likely to compress or grow into the sinus cavity to cause partial or even complete occlusion of the venous sinus.

For postcontrast radiological features, high-grade meningiomas more frequently showed heterogeneous enhancement (*p* = 0.005) but less presence of capsular enhancement (*p* = 0.041) and a dural tail sign (*p* = 0.011), in comparison with benign meningiomas. Other features were not found to be statistically different between the two types of tumors. Patients’ demographic and radiological features are summarized in **Table 3**. We evaluated the inter-observers’ variances using inter-class correlation coefficient (ICC) methods, and the results were convincing (with an ICC interval from 0.76 to 0.88).

**Table 3:** Demographic and radiological characteristics in the development cohort.

	Low grade	High grade	*p*-Value
**Number**	85	52	
**Age (years)**	57.33 ± 10.07	53.29 ± 12.39	0.064
**Sex**			
Male	18 (21.2%)	20 (38.5%)	0.028
Female	67 (78.8%)	32 (61.5%)	
**Tumor location**			
Convexity	32 (37.6%)	19 (36.5%)	0.837
Skull base	22 (25.9%)	15 (28.8%)	
Falx	16 (18.8%)	8 (15.4%)	
Posterior fossa	13 (15.3%)	7 (13.5%)	
Other	2 (2.4%)	3 (5.8%)	
**Volume** (×10^4^ mm^3^)	2.64 (1.03–7.09)	7.23 (3.70–14.31)	**<0.001**
**Shape**			
Regular	64 (75.3%)	11 (21.2%)	**<0.001**
Lobulated	15 (17.6%)	15 (28.8%)	
Irregular	6 (7.1%)	26 (50.0%)	
**T2 signal feature**			
Heterogeneous	46 (54.1%)	42 (80.8%)	**0.002**
Homogeneous	39 (45.9%)	10 (19.2%)	
**T2 signal intensity**			
Hypointense	1 (1.2%)	3 (5.8%)	0.372
Isointense	12 (14.1%)	6 (11.5%)	
Hyperintense	72 (84.7%)	43 (82.7%)	
**Cystic change**			
Yes	13 (15.3%)	27 (51.9%)	**<0.001**
No	72 (84.7%)	25 (48.1%)	
**Brain edema** (mm)	0.00 (0.00–12.75)	21.50 (3.83–39.85)	**<0.001**
**≤20**	72 (84.7%)	25 (48.1%)	**<0.001**
**20 < edema ≤ 40**	10 (11.8%)	13 (25.0%)	
**>40**	3 (3.5%)	14 (26.9%)	
**Tumor–brain interface**			
Clear	85	51	0.380
Unclear	0	1	
**nADC**	1.07 (0.98–1.21)	1.03 (0.89–1.19)	0.206
**Contrast enhancement**			
Heterogeneous	51 (60.0%)	43 (82.7%)	**0.005**
Homogeneous	34 (40.0%)	9 (17.3%)	
**Capsular enhancement**			
Present	20 (23.5%)	5 (9.6%)	**0.041**
Not present	65 (76.5%)	47 (90.4%)	
**Dural tail sign**			
Present	70 (82.4%)	31 (59.6%)	**0.003**
Not present	15 (17.6%)	21 (40.4%)	

Bold values indicate p < 0.05.

### Performance of clinicoradiologic model and nomogram

**Figure 2:**
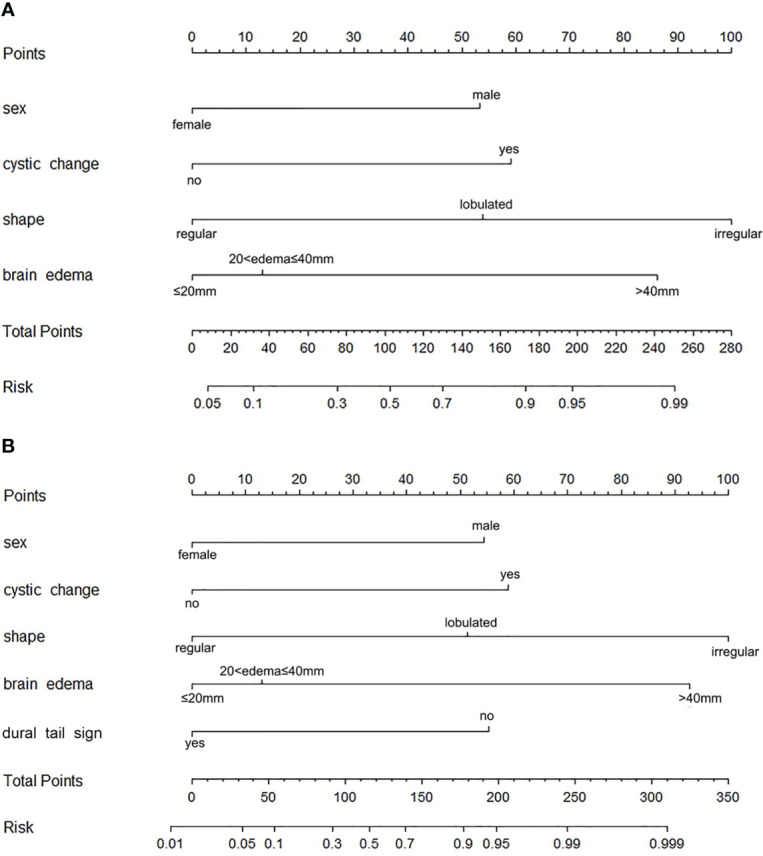
Nomograms for identifying high-grade meningiomas. **(A)** The precontrast–postcontrast nomogram was developed incorporating sex, intratumoral cystic changes, shape, brain edema, and dural tail sign. **(B)** The precontrast nomogram was developed incorporating sex and precontrast radiological features, including intratumoral cystic changes, shape, and brain edema.

**Figure 3:**
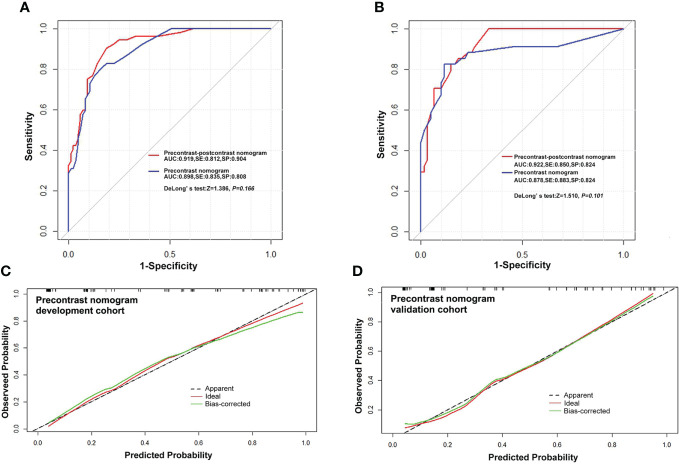
Receiver operating characteristic curves and calibration curves. **(A, B)** ROC curves for predicting high-grade meningiomas in the development and validation cohort with the precontrast–postcontrast model and precontrast model, respectively. **(C, D)** Curves of the calibration analysis for the precontrast model in the development and validation cohorts, respectively. The calibration curves of the precontrast–postcontrast model also showed a satisfactory calibration performance in both the development and validation cohorts (see **Supplementary Material**). ROC, receiver operating characteristic curve; AUC, area under ROC curve; SE, sensitivity; SP, specificity.

The precontrast–postcontrast model showed that male sex, intratumoral cystic changes, lobulated and irregular shape, brain edema, and absence of a dural tail sign were independent predictive factors for high-grade meningiomas (**Table 4**). The precontrast–postcontrast nomogram model was built (**Figure 2A**), and ROC curve analysis yielded an AUC of 0.92 (95% CI: 0.87–0.96), sensitivity of 0.81, and specificity of 0.90 in the development cohort and an AUC of 0.92 (95% CI: 0.87–0.97), sensitivity of 0.85, and specificity of 0.82 in the validation cohort. The precontrast model showed that male sex, intratumoral cystic changes, lobulated and irregular shape, and brain edema were independent predictive factors for high-grade meningiomas (**Table 4**). The precontrast nomogram model was built (**Figure 2B**), and ROC curve analysis yielded an AUC of 0.90 (95% CI: 0.85–0.95), sensitivity of 0.84, and specificity of 0.81 in the development cohort and an AUC of 0.88 (95% CI: 0.79–0.96), sensitivity of 0.88, and specificity of 0.82 in the validation cohort. DeLong’s test showed that there was no statistical difference between the two groups of ROC curves (ROC curves from the two models in the development cohort, Z = 1.386, *p* = 0.166; ROC curves from the two models in the validation cohort, Z = 1.510, *p* = 0.101). The ROC and calibration curves are displayed in **Figure 3**, and PR curves are provided in the **Supplementary Material**.

**Table 4:** The multivariable logistic regression analysis of two models.

	Precontrast–postcontrast model			Precontrast model		
	OR	95% CI	*p*-Value	OR	95% CI	*p*-Value
Male	6.91	2.07–23.04	0.002	5.25	1.72–16.03	0.004
Intratumoral cystic changes	8.14	2.36–28.11	0.001	6.27	2.03–19.33	0.001
Shape						
Lobulated shape	6.18	1.81–21.11	0.004	5.34	1.70–16.71	0.004
Irregular shape	34.96	7.93–154.17	<0.001	22.39	6.09–82.31	0.000
Brain edema (mm)						
20 < edema ≤ 40	1.59	0.38–6.61	0.523	1.50	0.42–5.39	0.537
>40	26.93	3.69–196.38	0.001	14.612	2.59–82.44	0.002
Dural tail sign	0.14	0.04–0.50	0.002			

Demographic features and precontrast and postcontrast MRI radiological features with p < 0.05 between two types of tumors.

**Figure 4:**
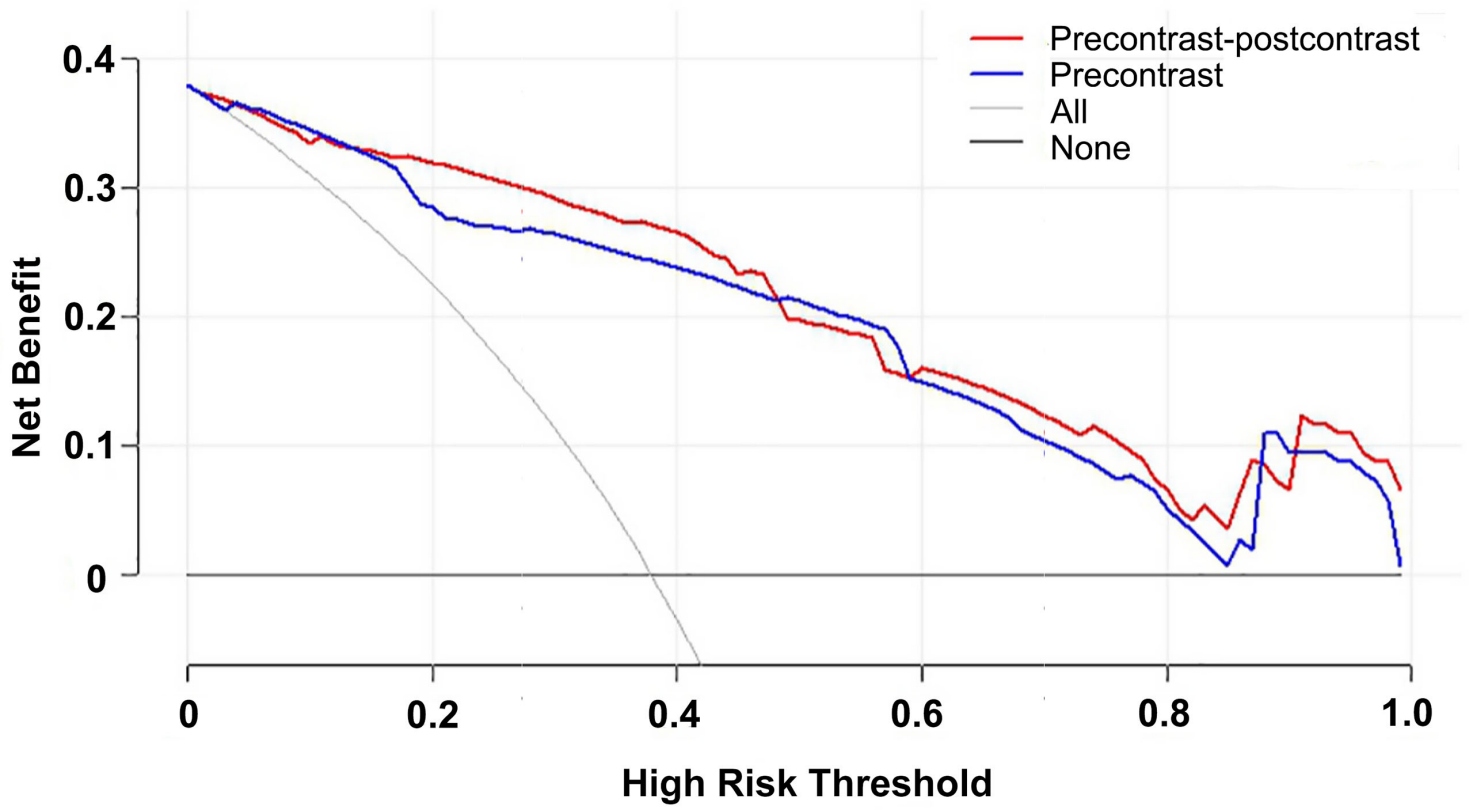
Decision curve analysis for the two nomograms. The net benefits of the precontrast–postcontrast nomogram were slightly superior to the benefits of the precontrast nomogram across most ranges of threshold probability. Red line, precontrast–postcontrast nomogram; blue line, precontrast nomogram; gray line, all patients were high grade; black line, all patients were low grade.

**Figure 5:**
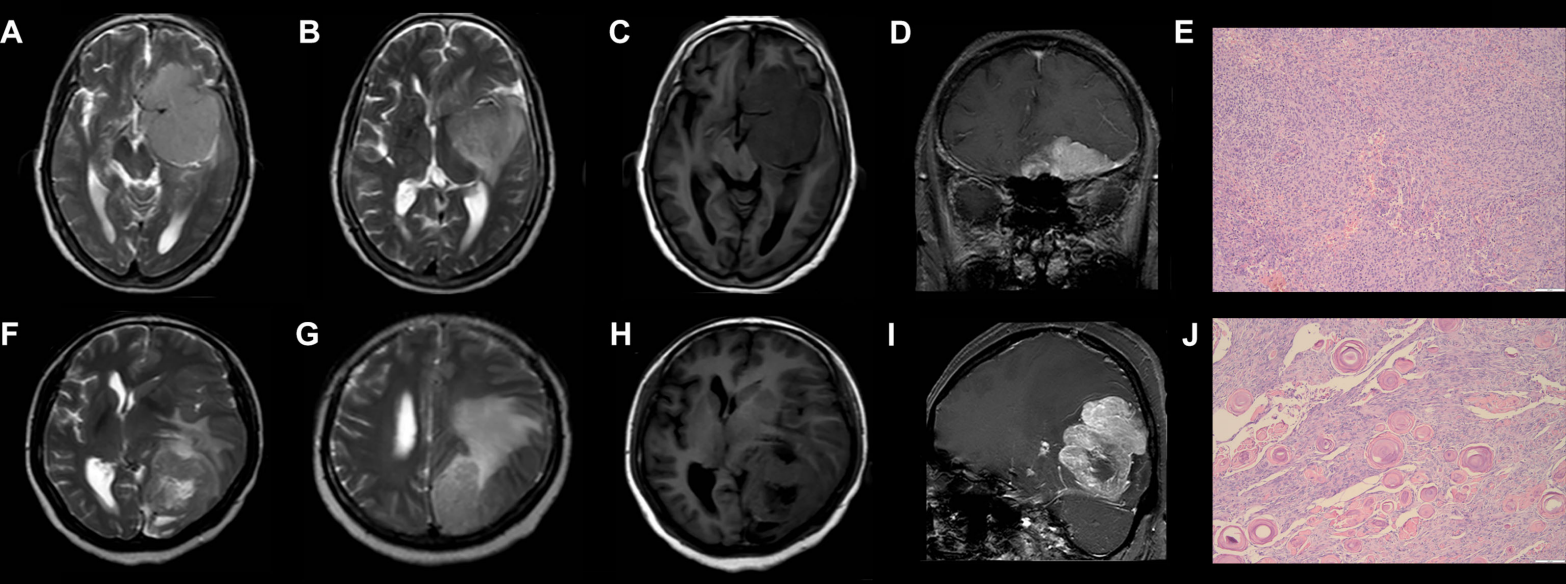
Examples of low-grade and high-grade meningiomas. **(A–E)** A 76-year-old woman who suffered from headache and hyposmia had a brain MRI examination. T2WI (A/B) and precontrast T1WI **(C)** show the tumor has a lobulated shape, homogeneous intensity without cystic change, and mild peritumoral brain edema (7.4 mm). Postcontrast T1WI **(D)** shows there is an obvious dural tail sign. According to the precontrast–postcontrast nomogram and the precontrast nomogram, the patient has total points of 50 and 55, respectively, and the risk probability of high-grade meningiomas is less than 0.1 and 0.2, respectively. She was histologically diagnosed with Grade I meningiomas **(E)**. **(F–J)** A 45-year-old woman who suffered from headache and hemianopia had a brain MRI examination. T2WI (F/G) and precontrast T1WI **(H)** show the tumor has a lobulated shape and intratumoral cystic change with large peritumoral brain edema (42.2 mm). Postcontrast T1WI **(I)** shows there is no obvious dural tail sign. According to the precontrast–postcontrast nomogram and the precontrast nomogram, the patient has total points of 257.5 and 200, respectively, and the risk probability of high-grade meningiomas is higher than 0.99 and 0.95, respectively. She was histologically diagnosed with Grade II meningiomas **(J)**. T2WI, T2-weighted imaging; T1WI, T1-weighted imaging.

The DCAs based on the two models suggested that the net benefits of the precontrast–postcontrast nomogram were slightly superior to the benefits of the precontrast nomogram across most ranges of threshold probability (see **Figure 4**). The examples of low-grade and high-grade meningiomas are displayed in **Figure 5**.

## Discussion

As treatment strategies are quite different between low-grade and high-grade meningiomas, it is always preferable to separate them before resorting to a biopsy or resection. This study has developed and validated the distinguishing performance of an easily used risk-scoring model to separate them using clinical characteristics and radiological features of precontrast MRI. Our results demonstrate that the risk-scoring model is qualified for this distinguishing task and would facilitate the risk stratification and clinical decision-making of meningiomas.

### Interpretation of demographic and radiological features

Previous studies have also reported an increased risk for high-grade meningiomas associated with the male sex (10, 11). Evidence suggests that low-grade meningiomas have higher levels of progesterone receptor expression in comparison with malignant meningiomas (12–14). Furthermore, clinical and histopathological studies showed that progesterone receptor expression levels were inversely associated with WHO grade and recurrence (14, 15). Genetic studies also have shown that differential gene expression is limited to sex chromosomes in meningioma cells (16).

Intratumoral cystic changes on MRI are considered to represent rapid growth or invasive behavior of tumor cells, including cystic degeneration, direct secretion of fluid by tumor cells, ischemic necrosis, and absorption of intratumor hemorrhage (17, 18). Our results agree with previous studies that showed that meningioma cystic changes were associated with tumor grade (17, 19).

Peritumoral edema in meningiomas has been reported to be related to many factors, such as tumor size and histological subtypes (17, 18, 20, 21). One recent study showed a direct association between the peritumoral edema index and the mutational burden of meningiomas, along with the fact that high-grade meningiomas were more likely to present with larger edema (22). One study reported that peritumoral brain edema may depend on the aquaporin-4 (AQP4) expression level, which is now considered a biomarker reflecting tumor malignancy (23). However, some studies found no significant correlation between the histological grades of meningiomas and peritumoral edema (24, 25).

Tumors with lobulated or irregular shapes reflect heterogeneity in growth rates in different subregions of the tumor (26). Radiological features of lobulated or irregular shape may reflect histological malignancy. Studies have found that the lobulation ratio of meningiomas increases with the increase of malignant degree (27).

The presence of a dural tail sign is caused by the slow growth of low-grade meningiomas, which triggers an inflammatory reaction or long-term stimulation of the adjacent meninges, causing dural thickening. In contrast, the absence of a dural tail sign is considered a predictor of high-grade meningiomas. A potential reason may be that atypical neoplasms grow rapidly, can be detected at an early stage, and lack long-term meningeal stimulation. Although a previous study reported that a dural tail sign could also be found in high-grade meningiomas, the morphology of the dural tail is similar to that of a nodule in these high-grade meningiomas (26).

Values of ADC in distinguishing low-grade and high-grade tumors are controversial among previous studies (27, 28). The following reasons may explain this disagreement. Firstly, although there is an increase in the number of tumor cells, tumor stroma, fibers, or glial tissue may lead to a decrease in the ADC value of high-grade meningiomas. However, microscopic focal necrosis of atypical/malignant tumors may result in elevated water diffusion and may not be evident on MR images, thus increasing the ADC value of these high-grade lesions (29). Secondly, some subtypes of high-grade meningiomas such as chordoid meningiomas usually present with high ADC values, which may be related to the extracellular hyaluronic acid and mucinous matrix (30). Therefore, whether the mean ADC value could be a predictor of meningiomas malignancy requires further exploration.

Heterogeneous enhancement is associated with the heterogeneous distribution of tumor cells. Our results indicated that capsular enhancement was more frequent within low-grade meningiomas. As a previous study showed, this morphological character associated with a chronic reaction produces a more extensive external fibrous layer in benign, slower-growing tumors (31). The big volume associated with high-grade meningiomas, as shown in a previous study, may be attributed to the relatively high proliferative potential of high-grade tumors (32).

### Influence of clinicoradiologic model and nomogram

*Via* a nomogram model, a kind of risk-scoring model weighting each parameter in accordance with their regression coefficients in the multivariate logistic regression analysis, our results suggest that precontrast MRI is sufficient in identifying high-grade meningiomas. This risk-scoring model is easy to obtain with high robustness and generalizability because these radiological features are associated with certain pathophysiological meanings and are not sensitive to the variations of MR equipment. This model is helpful for reducing not only the scanning time and cost of MRI but also the potential risk of gadolinium contrast agents.

### Limitations

Our study has a few limitations. Firstly, this retrospective study only included a limited number of patients with Grade III anaplastic meningiomas. Due to the small number, the subtype difference between Grades II and III meningiomas was not analyzed. Secondly, the performance of our model was not compared with that of radiomics using the same MRI data; further study might be required to investigate the distinguishing difference between the two different methods. Thirdly, recent studies found that DNA-methylation status greatly influences the malignancy and prognosis of meningiomas, and the correlation between DNA-methylation status and selected radiological features was not explored. Further studies are needed in order to investigate these facts.

## Conclusions

In conclusion, male sex, intratumoral cystic changes, lobulated or irregular shape, and brain edema derived from commonly available precontrast MRI are independent predictors of high-grade meningiomas. An accessible risk-scoring system combined with these features shows good distinguishing performance and generalizability. This work would facilitate the risk stratification and clinical decision-making of meningiomas.

## Data availability statement

The raw data supporting the conclusions of this article will be made available by the authors, without undue reservation.

## Ethics statement

The studies involving human participants were reviewed and approved by Ethics committee on scientific research of Shandong University, Qilu Hospital. Written informed consent for participation was not required for this study in accordance with the national legislation and the institutional requirements. Written informed consent was obtained from the individual(s) for the publication of any potentially identifiable images or data included in this article.

## Author contributions

JH: Study conception and design, Critical revision. YY, YX: Study conception and design, Acquisition of data, Analysis and interpretation of data, Drafting of manuscript. SL, FX, SQ: Acquisition of data. BW, XS: statistical analysis. All authors contributed to the article and approved the submitted version.

## Funding

This study was supported by the Shandong Provincial Natural Science Foundation (ZR2021MH237 and ZR2021QH125).

## Acknowledgments

Thanks to Dmytro Pylypenko for the correction and proofreading of the article.

## Conflict of interest

The authors declare that the research was conducted in the absence of any commercial or financial relationships that could be construed as a potential conflict of interest.

## Publisher’s note

All claims expressed in this article are solely those of the authors and do not necessarily represent those of their affiliated organizations, or those of the publisher, the editors and the reviewers. Any product that may be evaluated in this article, or claim that may be made by its manufacturer, is not guaranteed or endorsed by the publisher.

## Abbreviations

ADC, apparent diffusion coefficient; AQP4, aquaporin-4; AUC, area under the ROC curve; CI, confidence interval; CSF, cerebrospinal fluid; DCA, decision curve analysis; MRI, magnetic resonance imaging; OR, odds ratio; ROI, region of interest; WHO, World Health Organization.

## References

[1] Ostrom QT Cioffi G Waite K Kruchko C Barnholtz-Sloan JS. CBTRUS statistical report: Primary brain and other central nervous system tumors diagnosed in the united states in 2014-2018. Neuro Oncol (2021)0) 23:i1–105. doi: 10.1093/neuonc/noab200 PMC8491279 34608945

[2] Louis DN Perry A Reifenberger G von Deimling A Figarella-Branger D Cavenee WK. The 2016 world health organization classification of tumors of the central nervous system: A summary. Acta Neuropathol (2016) 131:803–20. doi: 10.1007/s00401-016-1545-1 27157931

[3] Essig M Anzalone N Combs SE Dorfler A Lee SK Picozzi P. MR imaging of neoplastic central nervous system lesions: Review and recommendations for current practice. AJNR Am J Neuroradiol (2012) 33:803–17. doi: 10.3174/ajnr.A2640 PMC7968800 22016411

[4] Swaminathan S. Gadolinium toxicity: Iron and ferroportin as central targets. Magn Reson Imaging (2016) 34:1373–6. doi: 10.1016/j.mri.2016.08.016 27580520

[5] Mathur M Jones JR Weinreb JC. Gadolinium deposition and nephrogenic systemic fibrosis: A radiologist's primer. Radiographics (2020) 40:153–62. doi: 10.1148/rg.2020190110 31809230

[6] Joo B Han K Choi YS Lee SK Ahn SS Chang JH. Amide proton transfer imaging for differentiation of benign and atypical meningiomas. Eur Radiol (2018) 28:331–9. doi: 10.1007/s00330-017-4962-1 PMC5746026 28687916

[7] Bohara M Nakajo M Kamimura K Yoneyama T Fukukura Y Kiyao Y. Histological grade of meningioma: Prediction by intravoxel incoherent motion histogram parameters. Acad Radiol (2020) 27:342–53. doi: 10.1016/j.acra.2019.04.012 31151902

[8] Park YW Oh J You SC Han K Ahn SS Choi YS. Radiomics and machine learning may accurately predict the grade and histological subtype in meningiomas using conventional and diffusion tensor imaging. Eur Radiol (2019) 29:4068–76. doi: 10.1007/s00330-018-5830-3 30443758

[9] Figarella-Branger D Appay R Metais A Tauziede-Espariat A Colin C Rousseau A. [the 2021 WHO classification of tumours of the central nervous system]. Ann Pathol (2022) 42:367–82. doi: 10.1016/j.annpat.2021.11.005 34865882

[10] Kane AJ Sughrue ME Rutkowski MJ Shangari G Fang S McDermott MW. Anatomic location is a risk factor for atypical and malignant meningiomas. Cancer-Am Cancer Soc (2011) 117:1272–8. doi: 10.1002/cncr.25591 PMC3795515 21381014

[11] Mahmood A Caccamo DV Tomecek FJ Malik GM. Atypical and malignant meningiomas: A clinicopathological review. Neurosurgery (1993) 33:955–63. doi: 10.1227/00006123-199312000-00001 8134008

[12] Korhonen K Salminen T Raitanen J Auvinen A Isola J Haapasalo H. Female predominance in meningiomas can not be explained by differences in progesterone, estrogen, or androgen receptor expression. J Neurooncol (2006) 80:1–7. doi: 10.1007/s11060-006-9146-9 16703453

[13] Whittle IR Foo MS Besser M Vanderfield GK. Progesterone and oestrogen receptors in meningiomas: Biochemical and clinicopathological considerations. Aust N Z J Surg (1984) 54:325–30. doi: 10.1111/j.1445-2197.1984.tb05327.x 6593026

[14] Wolfsberger S Doostkam S Boecher-Schwarz HG Roessler K van Trotsenburg M Hainfellner JA. Progesterone-receptor index in meningiomas: Correlation with clinico-pathological parameters and review of the literature. Neurosurg Rev (2004) 27:238–45. doi: 10.1007/s10143-004-0340-y 15168138

[15] Hsu DW Efird JT Hedley-Whyte ET. Progesterone and estrogen receptors in meningiomas: Prognostic considerations. J Neurosurg (1997) 86:113–20. doi: 10.3171/jns.1997.86.1.0113 8988089

[16] Tabernero MD Espinosa AB Maillo A Rebelo O Vera JF Sayagues JM. Patient gender is associated with distinct patterns of chromosomal abnormalities and sex chromosome linked gene-expression profiles in meningiomas. Oncologist (2007) 12:1225–36. doi: 10.1634/theoncologist.12-10-1225 17962616

[17] Hale AT Wang L Strother MK Chambless LB. Differentiating meningioma grade by imaging features on magnetic resonance imaging. J Clin Neurosci (2018) 48:71–5. doi: 10.1016/j.jocn.2017.11.013 29174756

[18] Hsu CC Pai CY Kao HW Hsueh CJ Hsu WL Lo CP. Do aggressive imaging features correlate with advanced histopathological grade in meningiomas? J Clin Neurosci (2010) 17:584–7. doi: 10.1016/j.jocn.2009.09.018 20219376

[19] Zhou JL Liu JL Zhang J Zhang M. Thirty-nine cases of intracranial hemangiopericytoma and anaplastic hemangiopericytoma: A retrospective review of MRI features and pathological findings. Eur J Radiol (2012) 81:3504–10. doi: 10.1016/j.ejrad.2012.04.034 22658867

[20] Lin BJ Chou KN Kao HW Lin C Tsai WC Feng SW. Correlation between magnetic resonance imaging grading and pathological grading in meningioma. J Neurosurg (2014) 121:1201–8. doi: 10.3171/2014.7.JNS132359 25148010

[21] Zhang S Chiang GC Knapp JM Zecca CM He D Ramakrishna R. Grading meningiomas utilizing multiparametric MRI with inclusion of susceptibility weighted imaging and quantitative susceptibility mapping. J Neuroradiol (2020) 47:272–7. doi: 10.1016/j.neurad.2019.05.002 PMC6876125 31136748

[22] Gill CM Loewenstern J Rutland JW Arib H Pain M Umphlett M. Peritumoral edema correlates with mutational burden in meningiomas. Neuroradiology (2021) 63:73–80. doi: 10.1007/s00234-020-02515-8 32789536

[23] Gawlitza M Fiedler E Schob S Hoffmann KT Surov A. Peritumoral brain edema in meningiomas depends on aquaporin-4 expression and not on tumor grade, tumor volume, cell count, or ki-67 labeling index. Mol Imaging Biol (2017) 19:298–304. doi: 10.1007/s11307-016-1000-7 27552812

[24] Gurkanlar D Er U Sanli M Ozkan M Sekerci Z. Peritumoral brain edema in intracranial meningiomas. J Clin Neurosci (2005) 12:750–3. doi: 10.1016/j.jocn.2004.09.029 16165364

[25] Pistolesi S Fontanini G Camacci T De Ieso K Boldrini L Lupi G. Meningioma-associated brain oedema: The role of angiogenic factors and pial blood supply. J Neurooncol (2002) 60:159–64. doi: 10.1023/a:1020624119944 12635663

[26] Liu H Zhou J Li W Liu G. Comparative analysis of the magnetic resonance imaging features between anaplastic meningioma and atypical meningioma. J Craniofac Surg (2016) 27:e229–33. doi: 10.1097/SCS.0000000000002361 27100633

[27] Sacco S Ballati F Gaetani C Lomoro P Farina LM Bacila A. Multi-parametric qualitative and quantitative MRI assessment as predictor of histological grading in previously treated meningiomas. Neuroradiology (2020) 62:1441–9. doi: 10.1007/s00234-020-02476-y 32583368

[28] Surov A Ginat DT Sanverdi E Lim C Hakyemez B Yogi A. Use of diffusion weighted imaging in differentiating between maligant and benign meningiomas. a multicenter analysis. World Neurosurg (2016) 88:598–602. doi: 10.1016/j.wneu.2015.10.049 26529294

[29] Pavlisa G Rados M Pazanin L Padovan RS Ozretic D Pavlisa G. Characteristics of typical and atypical meningiomas on ADC maps with respect to schwannomas. Clin Imaging (2008) 32:22–7. doi: 10.1016/j.clinimag.2007.07.007 18164390

[30] Baal JD Chen WC Solomon DA Pai JS Lucas CH Hara JH. Preoperative MR imaging to differentiate chordoid meningiomas from other meningioma histologic subtypes. AJNR Am J Neuroradiol (2019) 40:433–9. doi: 10.3174/ajnr.A5996 PMC6420369 30819773

[31] Kawahara Y Nakada M Hayashi Y Kai Y Hayashi Y Uchiyama N. Prediction of high-grade meningioma by preoperative MRI assessment. J Neurooncol (2012) 108:147–52. doi: 10.1007/s11060-012-0809-4 22327898

[32] Hwang WL Marciscano AE Niemierko A Kim DW Stemmer-Rachamimov AO Curry WT. Imaging and extent of surgical resection predict risk of meningioma recurrence better than WHO histopathological grade. Neuro Oncol (2016) 18:863–72. doi: 10.1093/neuonc/nov285 PMC4864259 26597949

