# Supplementary material for: Predicting the grade of meningiomas by clinical–radiological features: A comparison of precontrast and postcontrast MRI
Source: Front Oncol. 2022 Dec 1;12:1053089. doi: 10.3389/fonc.2022.1053089 (PMC9752076; doi:10.3389/fonc.2022.1053089)

**Supplementary materials**

**1. Supplementary Table** MR scan protocols

| Development cohort (Philips Achieva 3.0T) | |
| --- | --- |
| Axial T1WI | FOV=230×230mm, voxel size 1.05×1.31×6 mm, TR = 2000 ms, TE = 10 ms |
| Axial T2WI | FOV=230×230mm, voxel size 1.05×1.31×6 mm, TR = 3000 ms, TE = 80 ms |
| Axial DWI | A single-shot, spin-echo echo-planar imaging sequence, b-values= 0, 1000 s/mm2 in 3 orthogonal directions, FOV=230×230mm, voxel size 1.2 × 1.73 ×6 mm, TR = 3000 ms, TE = 106 ms |
| T2-FLAIR | FOV=230×230mm, voxel size 0.85×1.12×6mm，TR=6000ms，TE=140ms, TI=2000ms |
| Postcontrast T1WI | Axial sagittal and coronal, intravenous contrast injection (gadopentetate dimeglumine 0.1 mmol/kg), voxel size 0.6×0.75×6mm，TR/TE=194/4.61 |
| Validation cohort (GE Discovery 750 3.0T) | |
| Axial T1WI | FOV=220×220mm, voxel size 0.9×0.9×5mm, TR = 2020 ms, TE = 18 ms |
| Axial T2WI | FOV=220×220mm, voxel size 0.7×0.7×5 mm, TR = 3400 ms, TE =109 ms |
| Axial DWI | A single-shot, spin-echo echo-planar imaging sequence, b-values= 0, 1000 s/mm2 in 3 orthogonal directions, FOV=220×220mm, voxel size 0.6×0.6×5mm, TR = 3200 ms, TE = 73 ms, |
| T2-FLAIR | FOV=220×220mm, voxel size 0.4×0.4×5mm，TR=8000ms，TE=111ms, TI=2500ms |
| Postcontrast T1WI | Axial sagittal and coronal, intravenous contrast injection (gadopentetate dimeglumine 0.1 mmol/kg), voxel size 0.9×0.9×5mm，TR/TE=2200/33 |

*T1WI, T1-weighted imaging; FOV, field of view, TR, time of repetition; TE, time of echo; T2WI, T2-weighted imaging; DWI, diffusion-weighted imaging; T2-FLAIR, T2-fluid attenuated inversion recovery; TI, time of inversion.*

**2. Supplementary Figure 1.** The calibration curves of the precontrast-postcontrast nomogram model in both the development and validation cohorts.


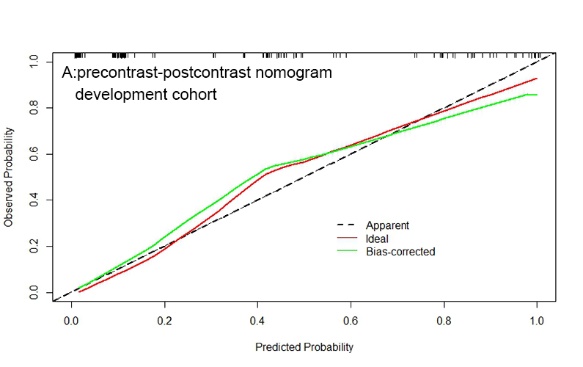

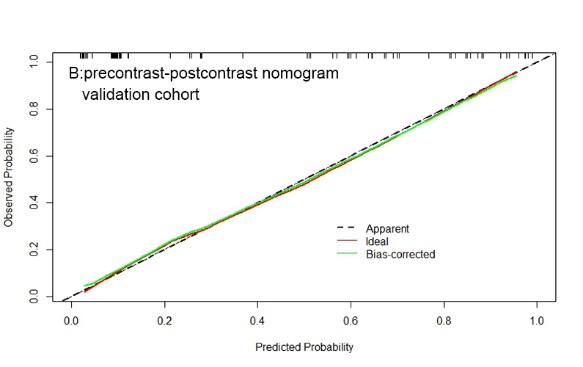


**3. Supplementary Figure 2.** The Precision-Recall curves in the development and validation cohort with the precontrast-postcontrast model and precontrast model, respectively.


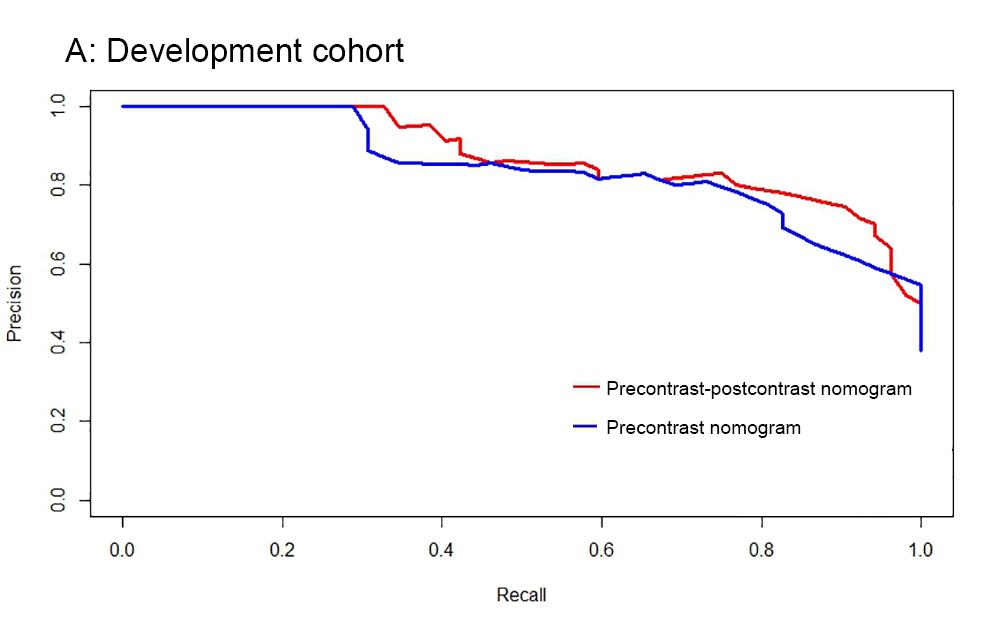

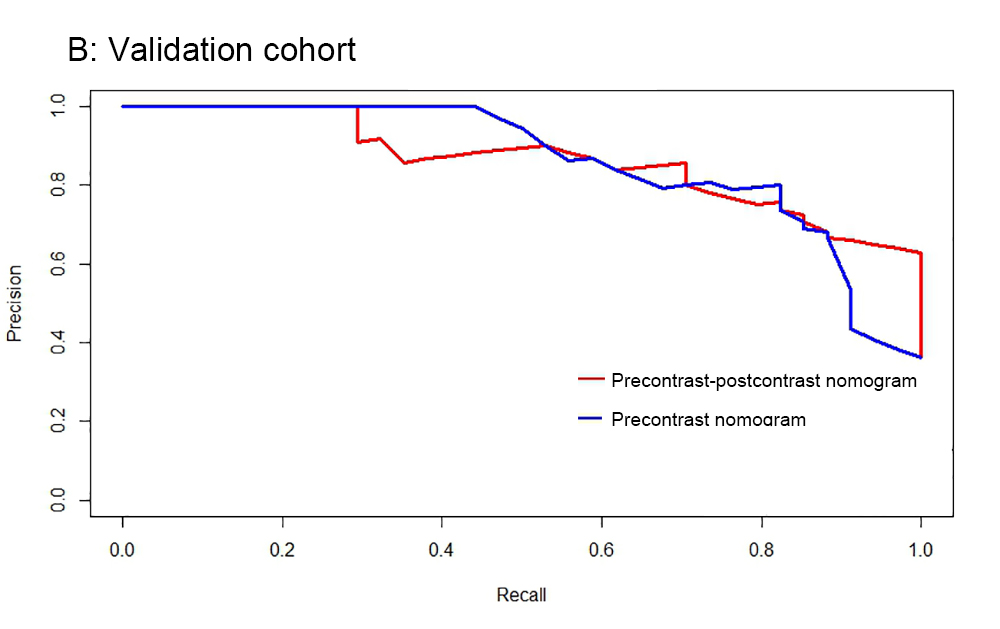

Supplement: Supplementary file 1 [file DataSheet_1.docx]
